# Evaluating the Effectiveness of a Web-Based Program (POP4Teens) to Prevent Prescription Opioid Misuse Among Adolescents: Randomized Controlled Trial

**DOI:** 10.2196/18487

**Published:** 2021-02-25

**Authors:** Lisa A Marsch, Sarah K Moore, Michael Grabinski, Sarah Y Bessen, Jacob Borodovsky, Emily Scherer

**Affiliations:** 1 Center for Technology and Behavioral Health Geisel School of Medicine at Dartmouth College Lebanon, NH United States; 2 HealthSim Inc Hanover, NH United States; 3 Square2 Systems, Inc Hanover, NH United States; 4 Geisel School of Medicine at Dartmouth College Hanover, NH United States; 5 Department of Biomedical Data Science Center for Technology and Behavioral Health Geisel School of Medicine at Dartmouth College Lebanon, NH United States

**Keywords:** opioids, prevention and control, adolescent, randomized controlled trial, internet

## Abstract

**Background:**

Prescription opioid (PO) use is common among adolescents in the United States. Despite recent declines from unprecedented peaks in adolescent PO use (eg, in 2012-2013), there is seemingly paradoxical evidence that PO-related consequences (eg, opioid use disorder and overdoses) are increasing. These trends and their possible consequences emphasize the importance of prevention efforts targeting PO misuse. To our knowledge, we have developed the first interactive web-based program (POP4Teens [P4T]) focused specifically on the prevention of PO misuse among adolescents.

**Objective:**

This study aimed to evaluate the effectiveness of P4T, a web-based program designed to prevent adolescent PO misuse, in comparison with JustThinkTwice (JTT), an active control website, on PO-related attitudes, knowledge, risk perception, and intentions to use.

**Methods:**

We conducted a web-based randomized controlled trial in 2018. A total of 406 adolescents (aged 12-17 years) were randomly assigned to either P4T or JTT. The outcome variables were attitudes, knowledge, and risk perceptions associated with PO misuse, intentions to use POs, and program feedback. Data were collected at baseline and at 1, 3, and 6 months.

**Results:**

Both programs resulted in significant and sustained improvements in intention to use POs, increased perceived risk, impacted expectancies consistent with prevention, and improved PO refusal skills. P4T produced significantly greater increases in PO-related knowledge than JTT did, and it was reportedly easier to use and more liked. Baseline scores for youth reporting past-year medical use of POs, friends who engage in nonmedical use of POs, and/or poor mental health underscored their at-risk status compared with youth from the other groups.

**Conclusions:**

P4T positively impacted all study variables that are known to prevent PO misuse among teens. Moreover, its web-based nature simplifies the dissemination and implementation of this novel tool designed to help meet the challenges of the evolving national opioid crisis.

**Trial Registration:**

ClinicalTrials.gov NCT02737696; https://clinicaltrials.gov/ct2/show/NCT02737696

## Introduction

### Background

Prescription opioid (PO) use is common among adolescents in the United States. Over the last 4 decades, the lifetime prevalence of medical (13%-20%) and nonmedical use of POs (NMUPO; 7%-13%) among high school seniors (approximately 17-18 years of age) has fluctuated yet both remain high [1]. One in every 100 adolescents aged between 12 and 17 years reported current NMUPO in 2017 [2], and this estimate may underreport the problem due to potential social desirability bias associated with self-report measures, particularly among youth [3]. Predictors of NMUPO and opioid use disorder (OUD) include early initiation of medical opioid use, and the risks of OUD are inversely correlated with age of onset [4-6]. Adolescents with mental health problems are also more likely than those without such problems to use diverted POs [7]. Adolescents with NMUPO overwhelmingly report accessing these medications from friends and family and having friends or family who are prescribed opioids increases the risk for misuse and OUD [8-10].

Compared with nonusers, adolescents who engage in NMUPO have increased odds of using alcohol and other drugs, engaging in criminal behaviors, using health care (eg, receiving emergency or inpatient treatment), receiving treatment services for mental health, and reporting poorer health [11-15]. The risk of transition to heroin use is greatest among those that begin NMUPO early in adolescence [16-19], and fatal and nonfatal overdose rates are higher for these youth compared with their peers who do not engage in NMUPO [20]. Despite recent declines from unprecedented peaks in adolescent PO use (eg, in 2012-2013), there is seemingly paradoxical evidence that PO-related consequences (eg, OUD, mortality, overdoses) are increasing [21,22]. These increases are likely associated with evidence that youth with OUD rarely receive the treatment they need [23].

These trends and their possible consequences emphasize the importance of prevention efforts targeting PO misuse. To our knowledge, we have developed the first interactive web-based program (POP4Teens [P4T]) focused specifically on the prevention of PO misuse among adolescents. This program extends our previous work to develop and evaluate science-based, computer-delivered substance misuse prevention programs for youth, including a prototype of this program we previously developed and evaluated [24-26]. The scientific evidence underpinning P4T’s program content comes from research on identified risk factors for PO use among youth [27], computer-delivered interventions [28], and computer-assisted instruction technology [29].

The P4T program is unique compared with other programs, as it is the first of its kind to incorporate knowledge about risk factors for PO misuse. PO misuse is unique compared with misuse of many other drugs: the threshold to access is low, potentiating the behavior (ie, usually obtained from friends and family and at no cost) [30,31]. Relatedly, youth who are prescribed opioids often divert their medication [32,33]; the perceived risks of misuse are significantly attenuated compared with other drugs, as they are dispensed by trusted medical providers [34,35]; youth perceive that parental consequences of misuse will be minor if they are *caught* with POs relative to other drugs [36].

In addition to a focus on the specific risk factors associated with POs, the interactive, activity-oriented P4T program is rooted in the prevention science literature, which has demonstrated that effective substance abuse prevention programs educate youth about the mechanism of action by which a substance produces its pharmacological effect and additionally include various types of skills training (eg, to resist peer, family, media, and other social influences known to promote drug use, and/or general skills training around social competency and coping with stressful life situations) [37,38]. Skills training increases protective factors, reduces risk factors, and produces reductions in drug use among youth [28]. Notably, programs based on social influence and general skills training models have been shown to be effective when delivered via interactive, activity-oriented programs and not traditional, didactic instructional techniques [39].

The P4T program is also unique as it is web-delivered and mobile-friendly and therefore available for use in a variety of settings. A recent scoping review of consumer health information technology (CHIT) in the prevention of substance use [40] underscores the effectiveness of *user-centered, interactive web-mediated apps* designed to *improve information access and exchange, decision making*, and *facilitate behavior changes that promote health and well-being*. Traditional means of reaching youth are hamstrung by costs associated with teacher or clinician-delivered interventions as well as manifold issues impeding intervention delivery fidelity. As of 2018, 94% of teens reported going on web daily and almost 95% reported mobile device usage and/or ownership [41]. Compared with traditional delivery methods, CHIT interventions delivered via digital platforms boast benefits of greater appeal to youth [28], reduced costs [36], standardized content delivery [36], potential for delivery across different settings (eg, on the web, home, community organizations, and health care providers’ offices) [37], and minimized training and/or clinical staff burden [42].

Published scientific literature on digital platforms for interventions targeting adolescent substance misuse is burgeoning [43,44]. Such programs have demonstrated effectiveness in 3 contexts: primary care [45], schools [44], and homes [46-48]. They target the prevention of different classes of drugs: alcohol [43,44], cannabis [45,49], cigarettes [50] and generally consist of interactive web-based activities that function to increase drug-related knowledge and shape user attitudes and normative beliefs about substance use in ways that promote abstinence or delayed onset of use [51]. The web-based context for the P4T program focuses on a new class of drugs (POs) previously unexplored in a digital format and additionally employs unique informational technologies that are effective in promoting relevant knowledge and skills. The P4T content uses information technologies to teach content to mastery via interactive and computer-assisted instruction (engaged when youth access quizzes) and enables exploration of the information and skills being taught in a video-based simulated environment [29,52,53].

### Objectives

The randomized controlled trial described herein focused on the prevention of PO misuse among youth. This web-based trial is designed to evaluate the effectiveness of the web-based PO misuse prevention program P4T, developed by our research group, compared with an active control website JustThinkTwice (Drug Enforcement Administration [DEA]) to impact attitudes about knowledge and perceptions of risk associated with misuse of POs as well as intentions to use and actual use of POs. Details of the development and formative evaluation of P4T have been published elsewhere [24].

## Methods

### Design

Participants were assigned at random using a nonlinear random number generator based on an AES block cipher in counter mode to either the experimental, web-based P4T program or the DEA’s JustThinkTwice (JTT) website (the active control condition). The study team was not blinded to randomization. However, due to the web-based nature of the trial (eg, recruitment, randomization, data collection) and limited-to-nonexistent research team contact with participants following randomization, bias was minimized.

The CONSORT (Consolidated Standards of Reporting Trials) checklist has been provided in Multimedia Appendix 1.

### Participants

Adolescent participants (12-17 years old) were recruited for study participation through Facebook and Google AdWords. This age range was selected to capture youth before a notable increase in risk of hazardous consequences of nonmedical PO use observed from age 12 years to 14 years [6,54], through the end of high school or before college. To be eligible, youth needed to be willing to use the study website to complete assessments and participate in the interventions, have access to a computer, a tablet, or a mobile device with an internet connection, and be able to hear audio.

From a user perspective, once an interested youth clicked on an ad, they were directed to the study *landing page*, which provided basic information about the study aims and clickable icons to *learn more*. Upon clicking on *learn more*, interested youth were offered the opportunity to receive a call from a research assistant by entering basic contact information (ie, name, email, parent’s name, email, phone number, and best time to be reached) that the research assistant used to make contact, answer additional questions, collect zip code data, email the consent form, and enable backend, automatic random assignment to a study condition. Once consent was established, the research assistant emailed the baseline assessment, which, once completed (as evidenced by a timestamp record on the program’s *dashboard* menu of participant activities), prompted the research assistant to send the first electronic gift card via email to the participant as compensation for their time spent in completing the assessment. Youth received US $20 for baseline, US $30 for 1-, 3-, and 6-month follow-ups and US $30 as bonus if all assessments were completed. Participants completed all assessments on the web in a secure manner, which allowed for greater anonymity and has been shown to result in more valid data compared with data collected via in-person interviews [55,56]. Delivering assessments via computer also ensured that questionnaire administration was more consistent, data processing was eliminated, and lag time between data collection and analyses was reduced. All procedures were approved by the Dartmouth College Committee for the Protection of Human Subjects.

### Procedures

The experimental program P4T is a newly developed intervention [24] designed to increase knowledge about POs and their risks and address social influences on PO use, such as developing skills to refuse offers or requests for POs. The program comprised 8 primary modules, and the information and skills emphasized in each module are delivered through 4 components: a storytelling component, an information lesson component, a skill-based video, and a quiz. The 8 module topics are (1) what are opioids? (2) misconceptions that opioids are safe and nonaddictive, (3) misconceptions that use of POs without a prescription is legal, (4) risks of PO misuse, (5) nonmedication alternatives for pain management, (6) refusing offers to misuse POs, (7) refusing requests by others for a PO prescribed to you, and (8) how to know if you or someone you may know may be addicted.

In each module, users were introduced to a different character based on real stories of youth in treatment for OUD. By clicking on the character icon, users were brought to an introductory screen displaying an exemplary quote from the youth’s story regarding PO use. Here, users could choose to listen to a recording of the story, read the story transcript, or view story highlights.

The program then allowed users to move between the introductory storytelling screen and the remaining 3 components (lesson, video, and quiz) personalizing the navigational flow and contributing to the interactive nature of P4T. The lessons detailed the scientific underpinnings of the story and delivered information through an interactive carousel format. The videos provided participants with concrete methods to approach social interactions and dilemmas involving PO misuse. The videos employed an *action-reaction* model in which a behavioral scenario was shown, followed by one or more characters reflecting on the situation. Finally, the quizzes allowed program users to test their understanding of the module content. Youth were given a variable amount of time to respond to quiz questions, which are presented multiple times and worded slightly differently to increase fluency and mastery, depending on how fluent youth are with the material, for example, if they are repeatedly getting a question incorrect, they will be allowed more time to respond to the next time they see a version of the question. Thus, the quizzes further contributed to the interactive nature of the program. Participants were asked to complete the 8 modules over the course of 4 weeks (approximately 2 modules per week).

The active, educational control website JTT is a web-based educational initiative supported by the DEA, which provides science-based information about teen drug abuse prevention. The intended purpose of the website, according to a DEA press release on the occasion of the launch of the teen website reads that it is “a website especially for our teenagers that provides young people with straightforward information on the consequences of drug use and trafficking, including health, social, legal, and international consequences. The site does not inundate the user with DEA messages; rather the facts come straight from leading physicians, scientists, and legal experts.” [57]

The website is not strictly dedicated to POs, although opioids are well represented. People navigating the website will find drug information, news and media, true stories of youth who have suffered due to drug use, information about consequences of drug use, drug use facts and statistics, videos, and a brief quiz. Randomized youth were asked to use this website for 30 min twice per week for 4 weeks.

### Outcome Measures

Behavioral assessments accessible by links emailed to participants were completed at baseline and follow-up time points (1, 3, and 6 months). At baseline, participants completed a form measuring basic demographic and substance use history, for example, age, gender, race, ethnicity, grade in school, average school grades, lifetime prescriptions for opioids, past-year mental health status, substance use in general, PO use among participants and their friends, and past participation in PO abuse prevention programs. At the 1-month time point only, participants completed the program feedback survey. Given the pervasive misconceptions that many youths have in their understanding of the risks associated with PO misuse [58], we primarily focused on analyses of knowledge about PO misuse as well as measures that are highly predictive of future use (attitudes, intentions, skills, and perceived risk).

#### Primary

##### Attitudes Toward PO Misuse

Participants were asked about their expectancies associated with PO misuse. Positive expectancies (eg, reduce boredom) and negative expectancies (eg, get in trouble with parents) about such use were evaluated using a 5-point measure ranging from *strongly disagree* to *strongly agree*. Mounting evidence has suggested that outcome expectancies (expected or anticipated behavioral, cognitive, and emotional harms and benefits [59]) associated with substance use play a role in the initiation and progression of drug use [60]. We modeled these questions after expectancy items developed by researchers examining potential physical, social, and psychological consequence expectancies associated with alcohol consumption among youth [61]. Changes in expectancies appear to be an important target in substance use prevention across alcohol, tobacco, and marijuana [62].

##### Perceived Risk of PO Misuse

Participants were asked 2 separate questions about how much they think they risk harming themselves (1) physically and (2) in other ways if they try POs for nonmedical purposes (eg, 5-point scale ranging from *no risk* to *great risk*) [59]. These questions are used annually in the Monitoring the Future Study [63], and the reliability and validity of survey questions is extensively discussed in an *occasional paper* detailing design and procedures [64]. Other studies have also included questions modeled after survey questions [65]. Research has consistently shown that alcohol and cannabis use is less likely among youth who perceive those substances and/or substance use behaviors as risky for the user [66,67], and thus, perceived harmfulness is potentially central to early prevention strategies [68].

##### Knowledge About PO Abuse Prevention

Participants were asked to complete a 15-item, multiple-choice measure assessing knowledge about effective PO abuse prevention (eg, how opioids work in the body). Knowledge test questions were generated by the research team based on scientific literature associated with the 8 module topics in the P4T program (eg, nonmedication alternatives for pain management).

##### Skill Acquisition

Youth were asked to pretend a friend offered them a PO and estimate how hard it would be to refuse the offer (5-point scale of *very easy* to *very hard*) and their ability to refuse the offer (5-point scale of *definitely would* to *definitely would not*). The same format was used to ask youth to pretend a friend asked them to share or sell POs prescribed to them [69]. These items were modeled after drug resistance scales used in previous studies with youth of the same age [70,71].

#### Secondary

##### Assessment of Usage Data

We quantified usage statistics of the web-based program by assessing the extent of module completion.

##### Behavioral Intentions to Use Opioids Without a Prescription

Youth were asked about their intentions to use opioids without a prescription *even once or twice over the next 12 months* using a 5-point scale ranging from *definitely not* to *definitely will.* Those who provided an affirmative response indicating any likelihood were also asked about the likelihood of using opioids without a prescription nearly every month for the next 12 months. This 2-item composite (α=.73) was modeled after a measure used in a study [72] that found changes in marijuana expectations among a large cohort of adolescents associated with subsequent future marijuana initiations.

##### Feedback Survey

The feedback survey was a brief 15-item tool that included 11 visual analog scale items (Multimedia Appendix 2) and 4 open-ended response items (ie, What youth liked best? Least? Suggestions? Anything else to add?). Possible values for visual analog scale scores ranged from 0 to 100 mm and were anchored by the variable of interest probed in the item (eg, how interesting was the section of the program you just completed? score range: 0=not interesting to 100=very interesting).

### Data Analysis

Although random assignment was designed to account for baseline differences, groups were compared for differences in demographics and other characteristics using two-tailed *t* tests, Wilcoxon rank sum tests for nonnormal variables, and chi-square tests for categorical measures. Program feedback (collected at 1 month) was also compared via *t* tests. Primary analyses included all participants randomized to a study condition independent of early dropout, consistent with an intent-to-treat approach to clinical trials [73]. Analyses were performed using SAS statistical software (type I error=.05). Outcome measures were evaluated in separate analyses. Data from primary outcomes were evaluated using mixed effect models, which allow for nonindependent data within individuals and are robust to incomplete data. All participants with any baseline or follow-up assessments were included in the outcome analyses. In addition to the study condition indicator, these models included, in the fixed effects portion of the model, a main effect for time as well as a 2-way interaction between study condition and time. Before evaluating the comparative effectiveness of P4T, each program’s effectiveness in impacting primary outcome variables without comparison is reviewed, as neither has previously been evaluated. The key estimate from this model is the 2-way interaction effect, which indicates whether changes in outcome over time are different for the 2 conditions.

### Sample Size Calculation

In our previous work [74], a computer-based prevention intervention, relative to an efficacious life skills control condition, had a large impact on knowledge (Cohen *d*=0.84), a moderate impact on drug refusal (Cohen *d*=0.43), a small impact on drug use intentions and attitudes (Cohen *d*=0.20), and a moderate impact on alcohol use (Cohen *d*=0.37). Schwinn et al [69] found a comparable effect size for reducing substance use when comparing an internet-based prevention program with an assessment-only control group (Cohen *d*=0.40). The planned sample size of 400 participants was based on having sufficient power to detect group differences in all outcomes, assuming 80% retention at the 6-month follow-up [73,75,76]. Assuming baseline assessments account for at least 9% of the variance in corresponding outcomes (*r*=0.30), power is estimated to be greater than 99% using α=.05 (2 sided) for detecting a prevention group effect of Cohen *d*=0.84 on the outcome measure of knowledge. Power was estimated to be 92% using α=.05 (2 sided) for detecting a prevention group effect of Cohen *d*=0.37 on the outcome measure of substance use and 98% using α=.05 (2-sided) for detecting a prevention group effect of Cohen *d*=0.43 on the outcome measure of drug refusal. For the intention to use outcomes, we determined the sample size needed to detect Cohen *d*=0.30, which is slightly higher than the effect observed in previous work [74], to reflect the use of an active control group in the planned study instead of a comparison condition of demonstrated effectiveness. The power to detect that effect was 80% when the sample size was 320 (after 20% attrition) at the 6-month follow-up. 

## Results

### Overview

**Figure 1:**
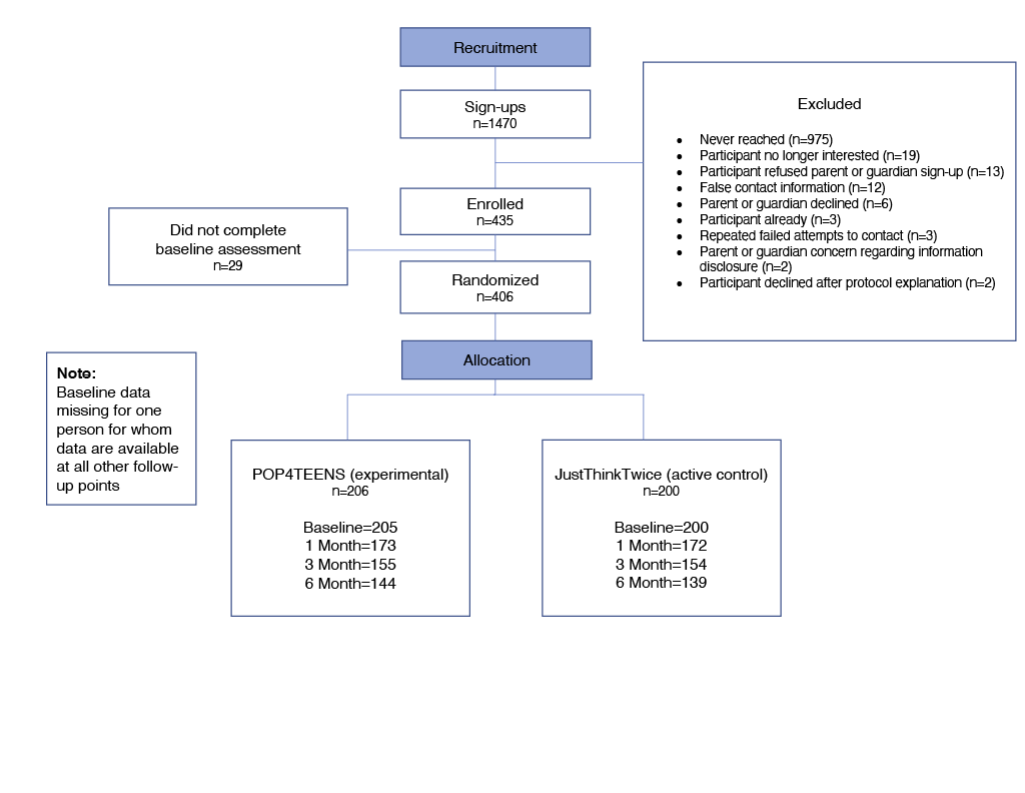
CONSORT (Consolidated Standards of Reporting Trials) diagram.

**Figure 2:**
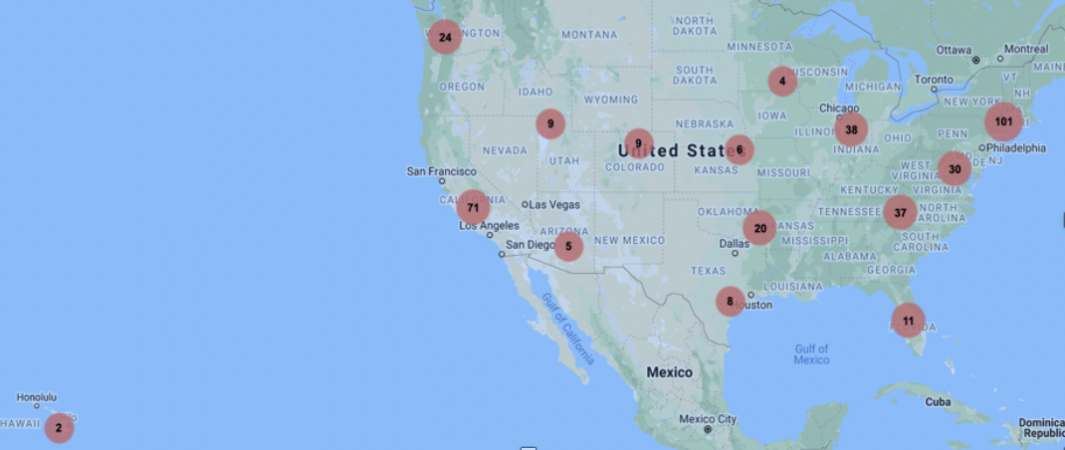
Geographic locations of participants. Zip code data for 379 of 406 participants (missing zip code data: n=27). In total, 41 of 50 states had at least one participant. Numbers in red circles are those of participants in a geographic area and may represent more than one state’s data. Alaska (n=4) is not shown on the map.

A nationwide sample of youth from 41 of the 50 US states was recruited between June 2017 and February 2018, and data collection was completed in September 2018. Of the 1470 youth and/or parent/guardian who signed up to gather more information about participation, 406 were consented and randomly assigned to one of the 2 websites (Figures 1 and 2).

### Participant Characteristics

The sample was primarily female (285/405, 70.4%), with a mean age of 15.8 (SD 1.2) years, and high achieving with 70.9% (287/405) reporting A’s for average school grade; 92.8% (376/405) reported good-to-excellent physical health, and approximately 73.6% (298/405) reported good-to-excellent mental health. More than a quarter of the sample reported lifetime exposure to medically prescribed opioids. Randomization was largely successful, as groups did not differ significantly on any baseline variable except for race: Asian (P4T/JTT: 21%/13.5%); Black/African American (P4T/JTT: 5.9%/12%); and White (P4T/JTT: 64%/67.5%; P=.03). For additional participant characteristics, see Table 1.

**Table 1:** Demographic and baseline characteristics.

Demographic and baseline characteristics			Overall (N=405)		P4T^a^ (n=205)		JTT^b^ (n=200)		P value
Age (years), mean (SE)			15.8 (1.2)		15.7 (1.2)		15.8 (1.2)		.80
**Gender, n (%)**									.22
	Female	285 (70.4)		148 (72.2)		137 (68.5)			
	Male	113 (27.9)		54 (26.3)		59 (29.5)			
	Transgender	5 (1.2)		1 (0.5)		4 (2)			
	No answer	2 (0.5)		2 (0.9)		0 (0)			
**Race, n (%)**									.03
	American Indian or Alaskan Native	3 (0.7)		1 (0.5)		2 (1)			
	Asian	70 (17.3)		43 (20.9)		27 (13.5)			
	Black or African American	36 (8.8)		12 (5.9)		24 (12)			
	Pacific Islander	4 (0.9)		4 (1.9)		0 (0)			
	White	266 (65.7)		131 (63.9)		135 (67.5)			
	Other	26 (6.4)		14 (6.8)		12 (6)			
**Ethnicity, n (%)**									.20
	Latino or Hispanic	49 (12.1)		29 (14.1)		20 (10)			
	Non-Latino or non-Hispanic	356 (87.9)		176 (85.9)		180 (90)			
**Grade, n (%)**									.27
	6th-9th (ages 13-15 years)	64 (15.8)		31 (15.1)		33 (16.5)			
	10th-12th (ages 16-18 years)	324 (80)		169 (82.4)		155 (77.5)			
	Not in school or other	17 (4.2)		5 (2.4)		12 (6)			
**Average school grade, n (%)**									.88
	A (90-100)	287 (70.9)		144 (70.2)		143 (71.5)			
	B (80-89)	100 (24.7)		52 (25.4)		48 (24)			
	C (70-79)	15 (3.7)		7 (3.4)		8 (4)			
	D (60-69)	2 (0.5)		1 (0.5)		1 (0.5)			
	F (<60)	1 (0.2)		1 (0.5)		0 (0)			
**Poor or fair mental health, n (%)**									.35
	No	298 (73.6)		155 (75.6)		143 (71.5)			
	Yes	107 (26.4)		50 (24.4)		57 (28.5)			
**Physical health, n (%)**									.19
	Excellent	93 (22.9)		50 (24.4)		43 (21.5)			
	Very good	177 (43.7)		85 (41.5)		92 (46)			
	Good	106 (26.2)		58 (28.3)		48 (24)			
	Fair	24 (5.9)		8 (3.9)		16 (8)			
	Poor	5 (1.2)		4 (1.9)		1 (0.5)			
**Ever prescribed opioids, n (%)**									.41
	Yes	106 (26.2)		50 (24.4)		56 (28)			
	No	299 (73.8)		155 (75.6)		144 (72)			
**Friends who use prescription opioids, n (%)**									.49
	Yes	68 (16.8)		37 (18)		31 (15.5)			
	No	337 (83.2)		168 (81.9)		169 (84.5)			
**Participated in a prescription opioid prevention program, n (%)**									.34
	Yes	11 (2.7)		4 (1.9)		7 (3.5)			
	No	394 (97.3)		201 (98)		193 (96.5)			

^a^P4T: POP4Teens.

^b^JTT: JustThinkTwice.

### Program Effectiveness (Within-Condition Effects)

#### Drug Use Outcome Expectancies (Attitudes)

The P4T program produced significant improvements from baseline across the follow-up time points (1, 3, and 6 months) for all positive expectancies of POs. The JTT program produced a similar pattern of results except for *reduce boredom* (baseline-1 month: P=.11) and *weight loss* (baseline-6 months: P=.06). The P4T program produced significant improvements from baseline across all follow-up time points for most negative expectancies. The exceptions were a pattern of nonsignificant results across all time points for *get in trouble with parents* and nonsignificance of *feel sleepy* (baseline-1 month: P=.41). The JTT program produced significant improvements from baseline across all follow-up time points for a few negative expectancies (eg, feel sleepy or overdose). The exceptions were a pattern of significant differences observed at the 1 and 6 month follow-ups, and nonsignificant differences at the 3-month follow-up (eg, spend too much money, feel sick, or pass out). The same pattern of nonsignificant differences across all time points for *get in trouble with parents* held for the JTT program. Finally, there were significant differences from baseline to 1 month that were not sustained at subsequent follow-ups for *do poorly in school.* For greater detail on within-condition effects, see Multimedia Appendix 2 (means) and Multimedia Appendix 3 (mixed models).

#### Perceived Risks

The P4T program significantly increased *perceived physical risks* from baseline to 1 month and baseline to 3 months; however, significant increases were not maintained at the third follow-up (P=.14). The program also significantly increased *perceived other risks* from baseline to 3 months; however, this effect was not observed for baseline to 1 month (P=.06) or baseline to 6 months (P=.06). The JTT program produced significant and sustained increases in perceived physical and other risks from baseline across all time points.

#### Knowledge

The P4T program significantly increased knowledge about POs from baseline to all time points. The JTT program produced no significant changes in knowledge at any follow-up time point.

#### Skills

The P4T program produced significant and sustained changes in the desired direction across all skill sets (ie, assessments of difficulty and ability to refuse offers and requests) and time points except for *ability to refuse an offer to misuse POs* at 3 months (P=.06). The JTT program produced significant and sustained changes in the desired direction across all skill sets except for *perceived difficulty to refuse an offer to misuse POs* at 1 month (P=.14).

#### Intentions

Both programs produced significant and sustained decreased intentions to use POs in the next 12 months across all time points.

#### Assessment of Usage

Participants in the P4T group completed an average of 6.43 (SD 2.98) of 8 possible modules and a total of 80.79% (1312/1624) possible modules. We were unable to calculate a similar metric in the control sample (JTT), as we did not control that website. However, we were able to calculate the average number of log-ins on the control site (a conservative estimate as participants may have chosen to access the site without using the portal on the study website [mean 4.06, SD 3.89]).

### Effectiveness of P4T Compared With JTT

#### Drug Use Outcome Expectancies (Attitudes)

There were no significant differences between groups for any positive expectancies at any time points. There were no significant differences between groups across the negative expectancies observed from baseline to 1 month. From baseline to 3 months, there were statistically significant increases in negative expectancies of POs favoring P4T: do poorly in school (P=.01), spend money (P=.01), feel sick (P=.03), and pass out (P=.004). From baseline to 6 months, the effect for do poorly in school was maintained (P=.01).

#### Perceived Risks

P4T produced a greater reduction (from baseline to 1 month) in the odds of perceiving a low *other* risk from POs compared with JTT (P=.03). There were no other significant differences between groups at any other time point for measures of risk.

#### Knowledge

There was a significant difference between groups in change from baseline for knowledge gains favoring P4T at the first 2 follow-up time points: baseline to 1 month (P<.001) and baseline to 3 months (P=.001). The effect somewhat diminished at the 6-month follow-up (P=.06).

#### Skills

There were no significant differences between groups across skills *except* for *difficulty refusing an offer to misuse* between baseline and 1 month (P=.05) favoring P4T. Differences were not maintained at later time points.

#### Intentions

There were no significant differences between groups in change from baseline to any of the 3 follow-up time points for *intention to use POs:* baseline to 1 month (P=.68), baseline to 3 months (P=.99), and baseline to 6 months (P=.75).

#### Feedback Surveys

With one exception, evaluations of the P4T and JTT programs were generally positive, with most mean scores at 7 or higher (scale 1-10) and not significantly different. However, the P4T program was rated as significantly easier to use than the JTT program (P<.001). In addition, the P4T youth liked using laptops, phones, and/or tablets significantly more to access the program than JTT youth (P<.001). No mean scores fell below 6, except for the applicability of program content to participants’ lives (Multimedia Appendices 2 [means] and 3 [mixed models]).

Four P4T program dimensions that youth liked best (in order of preference) were stories, ease of use, quizzes, and video. The stories were preferred for how they provided *real-life, relatable* examples and helped make *the opioid epidemic just seem more real.* Youth liked P4T because of how easy it was to *access* and *navigate.* They also reported enjoying the quizzes for how *the material began to make sense,* and the repetitive nature of the quiz questions helped youth *master* the information. Finally, some youth appreciated that the videos were upfront with being *a little bit corny at times* and were a *great educational tool.* The functionality of quizzes and videos surfaced among program dimensions was the least. Specifically, the timed and repetitive nature of the quizzes surfaced for critique:

The timed portion of the quizzes created unnecessary stress, increasing speed of the questions…lead to testing for your reaction time rather than processing information, the questions seemed very repetitive.

Videos were least liked by some because they were found to be *dull.*

### Baseline Differences: Characterizing the Sample in Terms of At-Risk Subgroups

#### Drug Use Outcome Expectancies (Attitudes)

Scores for youth reporting past-year medical use of POs, having friends who engage in NMUPO, and having poor mental health illuminated their at-risk status at baseline compared with youth who were not in those groups and therefore warrant attention. For negative expectancies, those with poor mental health agreed more strongly than those without poor mental health that overdose is likely when one engages in NMUPO. There was a pattern across those with a history of past year medical use of POs and having friends who engage in NMUPO that NMUPO would likely result in trouble with parents and doing poorly in school compared with those without such histories. In addition, those with a history of past year medical use of POs agreed more strongly that NMUPO would make one feel sleepy. For positive expectancies from POs, there is a clear pattern for both those with histories of poor mental health and having friends who engage in NMUPO demonstrating greater agreement with almost all such items compared with those without these histories, indicating an increased risk for these groups of youth. The only positive expectancy score for those with past year medical use of POs histories higher than their comparators was for reducing physical pain.

#### Perceived Risks, Knowledge, Skills, and Intentions

There were no differences between groups at risk (poor mental health, past year medical use of POs, and having friends who engage in NMUPO) and those not at risk in these categories for perceived risks with the lone exception among those with friends who engage in NMUPO who demonstrated slightly weaker agreement than those without friends who engage in NMUPO that there may be *other* risks (beyond physical), if one NMUPO. There were also no differences between youth in these 3 at-risk groups and their counterparts without such histories on knowledge about POs. Similar to the pattern of findings for positive expectancies, those with poor mental health and friends who engage in NMUPO scored at greater risk with respect to skills compared with participants without such histories. There were no differences between those with past year medical use of POs and those without such histories. Finally, all 3 subgroups reported stronger intentions to NMUPO in the next 12 months compared with youth not in those groups (Table 2).

**Table 2:** Primary and secondary measures of at-risk youth at baseline (mean).

Baseline value^a^			Poor mental health							Medical prescription opioid use							Friend who NMUPO^b^				
			Yes, mean (SD)		No, mean (SD)		P value		Yes, mean (SD)			No, mean (SD)		P value		Yes, mean (SD)			No, mean (SD)		P value
Intention to use prescription opioids			1.63 (0.77)		1.43 (0.69)		.02		1.62 (0.76)			1.44 (0.70)		.03		1.76 (0.92)			1.43 (0.66)		.01
**Negative expectancies**																					
	Spend too much (US $)	3.92 (1.07)		3.81 (1.11)		.41		3.73 (1.18)			3.88 (1.07)		.23		3.85 (1.20)			3.84 (1.08)		.93	
	Overdose	3.09 (1.13)		2.77 (1.11)		.01		2.87 (1.16)			2.86 (1.11)		.92		2.79 (1.10)			2.87 (1.13)		.60	
	Trouble with parents	4.58 (0.82)		4.53 (0.95)		.58		4.37 (0.99)			4.60 (0.88)		.03		4.19 (1.14)			4.61 (0.85)		.01	
	Pass out	3.44 (0.93)		3.30 (0.93)		.18		3.22 (0.98)			3.38 (0.92)		.15		3.24 (0.95)			3.35 (0.93)		.35	
	Do poorly in school	3.93 (0.99)		3.96 (1.09)		.79		3.70 (1.11)			4.04 (1.03)		.01		3.60 (1.15)			4.02 (1.03)		.01	
	Feel sick	3.79 (0.94)		3.80 (0.90)		.95		3.80 (0.97)			3.80 (0.88)		.98		3.75 (1.00)			3.81 (0.89)		.65	
	Feel sleepy	3.85 (0.89)		3.75 (0.90)		.34		3.95 (0.96)			3.72 (0.87)		.03		3.79 (0.99)			3.78 (0.88)		.89	
**Positive expectancies**																					
	Reduce anxiety	3.12 (1.17)		2.73 (1.05)		.003		2.86 (1.12)			2.82 (1.08)		.77		3.22 (1.08)			2.75 (1.08)		.003	
	Reduce boredom	2.45 (1.14)		2.10 (1.07)		.01		2.08 (1.11)			2.23 (1.10)		.23		2.49 (1.23)			2.14 (1.07)		.03	
	Escape problems	2.35 (1.10)		2.01 (1.01)		.01		2.18 (1.07)			2.07 (1.03)		.35		2.44 (1.15)			2.03 (1.01)		.01	
	Feel good	2.95 (1.04)		2.56 (1.07)		.001		2.84 (1.14)			2.60 (1.04)		.06		3.03 (1.15)			2.59 (1.04)		.004	
	Reduce physical pain	3.67 (1.10)		3.48 (1.04)		.12		3.81 (0.94)			3.43 (1.08)		.001		3.78 (1.08)			3.48 (1.05)		.04	
	Reduce sadness	2.76 (1.04)		2.44 (1.00)		.01		2.42 (0.99)			2.56 (1.03)		.24		2.76 (1.01)			2.47 (1.02)		.03	
	Improve social situations	2.31 (0.96)		1.93 (0.93)		<.001		2.02 (1.02)			2.03 (0.93)		.92		2.25 (1.06)			1.98 (0.92)		.06	
	Lose weight	2.57 (1.00)		2.45 (0.99)		.31		2.46 (1.05)			2.49 (0.97)		.79		2.51 (1.04)			2.48 (0.98)		.80	
**Perceived risks**																					
	Physical	4.28 (0.87)		4.37 (0.81)		.33		4.30 (0.95)			4.37 (0.78)		.53		4.22 (0.91)			4.38 (0.81)		.20	
	Other	4.35 (0.78)		4.44 (0.76)		.28		4.40 (0.82)			4.42 (0.74)		.77		4.21 (0.94)			4.46 (0.72)		.04	
**Skills**																					
	How hard to refuse offer	1.81 (0.95)		1.49 (0.77)		.002		1.58 (0.79)			1.58 (0.85)		.96		1.79 (0.96)			1.54 (0.80)		.04	
	Able to refuse offer	1.69 (0.78)		1.38 (0.72)		<.001		1.50 (0.88)			1.45 (0.70)		.59		1.69 (0.87)			1.42 (0.71)		.02	
	How hard to refuse request for prescription	1.88 (1.05)		1.52 (0.82)		.002		1.58 (0.85)			1.62 (0.92)		.62		1.93 (1.06)			1.55 (0.86)		.01	
	Able to refuse request for prescription	1.67 (0.89)		1.46 (0.80)		.03		1.60 (0.97)			1.49 (0.77)		.28		1.72 (0.96)			1.48 (0.80)		.06	
Knowledge		13.31 (2.00)		13.18 (2.18)		.58		13.32 (2.15)			13.18 (2.12)		.56		13.28 (2.30)			13.20 (2.09)		.80	

^a^Likert item anchors or values: expectancies: strongly disagree=1, disagree=2, not sure=3, agree=4, strongly agree=5; perceived risks: no risk=1, not sure=3, great risk=5; skills or difficulty: very easy=1, easy=2, not sure=3, hard=4, very hard=5; skills or ability: definitely would=1, would=2, not sure=3, would not=4, definitely would not=5; intentions: definitely will not=1, will not=2, not sure=3, will=4, definitely will=5.

^b^NMUPO: nonmedical use of prescription opioids.

## Discussion

### Principal Findings

To our knowledge, we developed the first interactive, web-based, and mobile-friendly program focused specifically on the prevention of PO misuse among adolescents. This study is the first to evaluate the effectiveness of this novel tool designed to help meet the challenges of the evolving opioid crisis in the United States.

The study yielded 4 key findings. First, the results demonstrate that both the P4T and the comparison JTT programs performed well in targeting variables known to impact NMUPO. Both programs were effective in steadily and significantly decreasing agreement with positive expectancies (in all cases for P4T/in most cases for JTT) and increasing agreement with negative expectancies potentially associated with PO use over time. The exception was the potential to get in trouble with parents, and yet the baseline scores for this item were already only a half-point from the full agreement, highlighting a ceiling effect limiting significant change. A recent longitudinal study of 3396 ethnically diverse high school students (9th and 10th grades) found that increases in positive substance use expectancies among those who never used each respective substance predicted increased odds of onset during late adolescence (11th and 12th grades; alcohol: odds ratio [OR]_ß_ 7.73, P<.001; tobacco: OR_ß_ 5.58, P<.001; marijuana: OR_ß_ 2.49, P=.001) [62]. Mounting evidence has suggested that outcome expectancies associated with substance use play a role in the initiation and progression of drug use [77]. Thus, change in expectancies is an important target in general substance use prevention and likely for PO misuse prevention as well.

Similar to the ceiling effect for the negative expectancy potential to get in trouble with parents, youth in both conditions perceived great risks at baseline; thus, limiting room for statistically significant change. However, both programs encouraged youth to increase their negative expectations around PO use. A systematic review of risk and protective factors associated with nonmedical use of prescription drugs (including opioids) among youth in the United States identified perceived risks as among the strongest and most consistent contributing factors at the individual level [78]. Both programs effectively impacted youth’s assessment of the difficulty and one’s ability to refuse offers to misuse POs as well as requests for personally prescribed POs. Scores across these variables were already close to optimal at baseline with little room for change. However, both programs produced statistically significant changes.

The second key finding is that P4T outperformed JTT on knowledge and negative expectancy changes as well as perceived ease of use, ease of understanding, and how much youth liked using technology to access the program. Knowledge-based, stand-alone interventions do not prevent later substance use. Programs that combine knowledge-based components with social competence and social influence approaches have greater support for their efficacy [79]. In addition, the JTT program focused on opioids among other drugs. However, it is significant that the program was effective in helping youth attain and sustain mastery of this important content.

The P4T youth also performed better than the JTT in increasing negative expectancy changes. Cross-sectional and longitudinal research findings indicate that the level of negative substance use expectancies is negatively associated with substance use behavior and use onset [80]. As noted above, although expectancy change and its effect on substance use onset may be more salient for positive expectancies (relative to negative), including both proximal and distal, negative expectancy items may increase the power of negative expectancies to impact substance use as powerfully as positive expectancies. The P4T program enlisted the stories of 8 youths detailing countless negative effects, both proximal and distal, of their experiences with NMUPO. It is possible that the level of detail with respect to negative effects through long periods of time (in most instances) served to more significantly impact the P4T scores in this area. The expectation-experience discrepancy (violating expectations) literature suggests that although it may seem reasonable in prevention efforts to stress the physical, psychological, and other harms of using drugs, this approach requires caution. Some marijuana-focused evidence suggests that if adolescents choose to use marijuana and the promised harms fall short of the severity they were led to expect; expectation changes may strengthen usage intentions [72]. However, legitimate opioid use before high school graduation is independently associated with a 33% increase in the risk of future opioid misuse after high school, and this association is concentrated among individuals who have little to no history of drug use and strong disapproval of illegal drug use at baseline [4]. It is highly plausible that expectation-experience discrepancies combined with the highly reinforcing properties of POs may have functioned to alter attitudes in this study.

Finally, the youth significantly preferred the P4T program to the JTT program in terms of ease of use cannot be underestimated. Drug prevention programs are arguably not at the top of the average adolescent’s list of preferred activities. Thus, lowering the barriers to a positive user experience in terms of program navigability and applicability to the lives of users is advantageous.

We drew a predominantly White, female, highly educated, middle adolescent (15-17 years), US sample, some of whom had personal histories of medical use of POs, friends who used POs nonmedically, and/or reported poor mental health. Aside from the highly educated [81,82] and White [83] characterizations of the sample, reporting past year medical use of POs, having friends who engage in NMUPO, and poor mental health are all often noted adolescent risk factors for NMUPO. The third key finding is that many youths in these latter 3 subgroups compared with youth not in the groups (comparators) scored consistently higher in terms of risk at baseline across variables of interest. Across all 3 subgroups (past year medical use of POs, having friends who engage in NMUPO, and poor mental health), intentions to use POs were significantly greater than those of comparators (P<.05). Youth with poor mental health and friends who engage in NMUPO underscored their elevated risk status by scoring almost all positive expectancies at a significantly higher rate than comparators, and perceived greater difficulty and lower ability to refuse offers and requests to use POs relative to comparators. These data support the growing evidence characterizing youth at greater risk for PO misuse. In addition, youth with past year medical use of POs and friends who engage in NMUPO tended toward disagreement with several negative expectancies relative to comparators (*trouble with parents* and *do poorly in school*). Those with a history of past year medical use of POs were in greater agreement than the comparators that POs might make a person *feel sleepy.* Thus, youth who had a history of past year medical use of POs may have more realistic expectations given their actual experience with the medications and be aware that POs do make you sleepy and that schoolwork does not have to suffer. Notably, youth who had past year medical use of Pos also scored only one positive expectancy (*reduce pain*) significantly higher relative to comparators. Consistent with the realistic expectations noted above, youth with medication experience may be aware that POs do indeed reduce pain. Although the evidence supports their at-risk status [4], the scores for youth who had past year medical use of POs function as a control on the other at-risk subgroups, as their differences from comparators are all consistent with having experienced the real consequences of past year medical use of POs.

The fourth key finding is that neither of the programs performed well in changing negative expectancy scores for *overdose*. Youth hovered around *not sure* across time points and programs for the negative expectancy of *overdose*, indicating that the risk of overdose does not seem to be a particularly salient concern in this sample. This is alarming and highlights an area where both programs need to do a better job of making this very real risk clearer. Between 1999 and 2016, the pediatric mortality rate from prescription and illicit opioids increased by 262.8% (N=approximately 9000) [84]. Annual rates were the highest for youth between 15 years and 19 years: 6755 (85.3%) of the deaths were unintentional, whereas 381 (4.8%) were attributed to suicide. In this group, the rates of fatal heroin, PO, and synthetic opioid poisonings increased by 404.8%, 94.7%, and 2925.0%, respectively. From 2014 to 2016, there were 1508 opioid deaths among adolescents between ages 15 and 19 years; of these, 468 (31.0%) were attributed to synthetic opioids (eg, fentanyl). One positive finding for one at-risk subgroup identified by our analyses was that youth with poor mental health scored significantly higher at baseline in agreement with the expectancy of overdose. Youth with poor mental health are at an increased risk of suicide [85]; thus, increasing awareness of the risks associated with NMUPO among this subgroup is reassuring. A 2018 survey of pediatric residents (N=69) examined knowledge, attitudes, and barriers to overdose prevention in the clinical setting. In total, 82% reported frequent exposure to pediatric patients using opioids and at risk of overdose. Although 94% of the trainees felt they had the responsibility to educate patients about overdose risk, only 42% discussed overdose prevention, and a mere 10% ever prescribed naloxone [86]. These findings reinforce that programs such as P4T and JTT must improve messaging around the potential for overdose when using POs medically and nonmedically.

### Limitations and Future Research

This study has several limitations. Recruitment advertisements were directed toward parents (after advertisements directed toward youth failed to yield interested families) skewing the sample in favor of youth from homes with parents attentive to substance misuse issues, that is, youth potentially less likely to have problems with substances or at least youth with concerned parents, impacting the generalizability of the sample. Participants were also not blinded; thus, knowledge of group assignment may have affected their behavior in the trial (eg, motivation to access the control group website) as well as their responses to subjective outcome measures. In addition, the knowledge questions posed to participants in both groups were derived from content in the P4T program and might have consequently inflated the differences between groups at multiple time points. Finally, although the measures used in this study have been used in previous studies, one limitation is that they have not been clinically validated.

Improving prevention focused on the risk factors for opioid misuse and OUD is one of the 4 central areas proposed by the National Institute on Drug Abuse, which is necessary for reversing the opioid crisis [87]. The US opioid crisis is now in its third wave [88] of opioid overdose-related deaths (wave 1: POs, wave 2: heroin, wave 3: synthetic opioids, eg, illicit fentanyl). As the nature of opioids driving the crisis evolves, prevention efforts will also need to evolve to respond effectively. On the basis of the results of our study, future research might include an enhanced approach to target at-risk subgroups (eg, poor mental health, having friends who engage in NMUPO, past year medical use of POs), as the pattern of findings point to different risk factors based on the different risk profiles (we plan to publish the differential effects of group assignment on at-risk subgroups). Moreover, due to the ceiling effect we found with respect to perceived physical and other risks and the floor effect for intentions to use, it may be useful to recruit youth who may already be experimenting and/or who endorse increased sensation-seeking or self-medicating profiles [89]. Recognizing the heterogeneity among NMUPO will enhance the evolution and efficient tailoring of prevention programs. Future research may also require an enhanced approach to target the misuse of synthetic opioids such as illicit fentanyl by youth, as underscored by the dramatic increase in death rates noted above. Another potential direction for future research is to attend to how parents contribute to or can protect against adolescent NMUPO. Parental NMUPO is associated with adolescent NMUPO [90], and parental use of opioids is associated with a doubling of the risk of a suicide attempt by their children (OR 1.99, 95% CI 1.71-2.33) [91]. However, parental involvement and disapproval of substance use [92] and monitoring as well as reduced conflict [90] are associated with reduced odds of lifetime NMUPO.

### Public Health Implications

The results suggest that the P4T program, which is freely available and accessible on the internet, may help prevent future misuse of PO among adolescents. Digital technology may provide scalable, effective prevention of PO misuse among teens.

## Appendix

Multimedia Appendix 1 CONSORT-EHEALTH checklist (V 1.6.1).

Multimedia Appendix 2 Primary and secondary outcome (means).

Multimedia Appendix 3 Primary and secondary outcome (mixed models).

## Abbreviations

CHIT: consumer health information technology
DEA: Drug Enforcement Administration
JTT: JustThinkTwice
NMUPO: nonmedical use of prescription opioids
OR: odds ratio
OUD: opioid use disorder
PO: prescription opioid
P4T: POP4Teens
