# Supplementary material for: Evaluating the Effectiveness of a Web-Based Program (POP4Teens) to Prevent Prescription Opioid Misuse Among Adolescents: Randomized Controlled Trial
Source: JMIR Public Health Surveill. 2021 Feb 25;7(2):e18487. doi: 10.2196/18487 (PMC8128362; doi:10.2196/18487)
Supplement: Multimedia Appendix 1 [file publichealth_v7i2e18487_app1.pdf]

CONSORT-EHEALTH Checklist V1.6.2 Report	Manuscript Number	18487

(based on CONSORT-EHEALTH V1.6), available at [http://tinyurl.com/consort-ehealth-v1-6].

Date completed

3/17/2020 16:09:43

by

Sarah K. Moore

Pop4Teens: Results of a randomized controlled trial to evaluate the effectiveness of a web-based program to prevent adolescent prescription opioid misuse

TITLE

1a-i) Identify the mode of delivery in the title

"POP4Teens: Results of a randomized controlled trial to evaluate the effectiveness of a web-based program to prevent adolescent prescription opioid misuse"

1a-ii) Non-web-based components or important co-interventions in title

There are none.

1a-iii) Primary condition or target group in the title

"POP4Teens...to prevent adolescent...misuse"

ABSTRACT

1b-i) Key features/functionalities/components of the intervention and comparator in the METHODS section of the ABSTRACT

Not included in the abstract due to space constraints.

1b-ii) Level of human involvement in the METHODS section of the ABSTRACT

1b-iii) Open vs. closed, web-based (self-assessment) vs. face-to-face assessments in the METHODS section of the ABSTRACT

1b-iv) RESULTS section in abstract must contain use data

1b-v) CONCLUSIONS/DISCUSSION in abstract for negative trials

INTRODUCTION

2a-i) Problem and the type of system/solution

"Prescription opioid use is not uncommon among adolescents in the United States... evidence that prescription opioid-related consequences are increasing. These trends and their possible consequences emphasize the importance of prevention efforts targeting PO misuse...we have developed the first interactive, web-based program focused specifically on prevention of PO misuse among adolescents... intended as a stand-alone intervention and for youth 12-17."

2a-ii) Scientific background, rationale: What is known about the (type of) system

"...digital platforms for interventions targeting adolescent substance misuse is burgeoning [43,44]. Such programs have demonstrated effectiveness in three contexts: primary care [45], schools [44] and homes [46-48]. They target the prevention of different classes of drugs... and generally consist of interactive, web-based activities that function to increase drug-related knowledge and shape user attitudes and normative beliefs around substance use in ways that promote abstinence or delayed onset of use [51]. The online context for the Pop4Teens program focuses on a new class of drugs (POs) previously unexplored in a digital format and additionally employs unique informational technologies which are effective in promoting relevant knowledge and skills... "

Does your paper address CONSORT subitem 2b?

"...to evaluate the effectiveness of the web-based prescription opioid misuse prevention program ...Pop4Teens compared to an active control website, JustThinkTwice.com (Drug Enforcement Administration) to impact attitudes about, knowledge and perceptions of risk associated with misuse of prescription opioids, as well as intentions to use and actual use of prescription opioids... "

METHODS

3a) CONSORT: Description of trial design (such as parallel, factorial) including allocation ratio

"Participants were assigned at random ... to either the experimental or the active control condition."

3b) CONSORT: Important changes to methods after trial commencement (such as eligibility criteria), with reasons

No important changes to methods after trial commencement.

3b-i) Bug fixes, Downtimes, Content Changes

4a) CONSORT: Eligibility criteria for participants

"...adolescents (12-17 yrs. eligible) ... To be eligible, youth needed to be willing to use the study website to complete assessments and participate in the interventions, have access to a computer with an Internet connection, and be able to hear audio."

4a-i) Computer / Internet literacy

4a-ii) Open vs. closed, web-based vs. face-to-face assessments:

This was a purely web-based trial.

"Participants were adolescents recruited for study participation through Facebook and Google AdWords... Upon clicking 'learn more,' interested youth were offered the opportunity to receive a call from a research assistant by entering basic contact information (i.e., name, email, parent's name, email, phone number and best time to be reached) that the research assistant used to make contact, answer additional questions, collect zip code data, email the consent form, and enable backend, automatic random assignment to a study condition...."

4a-iii) Information giving during recruitment

4b) CONSORT: Settings and locations where the data were collected

All data were collected online (see verbiage above in 4a-ii).

4b-i) Report if outcomes were (self-)assessed through online questionnaires

All data were collected online (see verbiage above in 4a-ii).

4b-ii) Report how institutional affiliations are displayed

5) CONSORT: Describe the interventions for each group with sufficient details to allow replication, including how and when they were actually administered

5-i) Mention names, credential, affiliations of the developers, sponsors, and owners

5-ii) Describe the history/development process

5-iii) Revisions and updating

5-iv) Quality assurance methods

5-v) Ensure replicability by publishing the source code, and/or providing screenshots/screen-capture video, and/or providing flowcharts of the algorithms used

5-vi) Digital preservation

5-vii) Access

Youth accessed the application online and received: "$20 for baseline, $30 for 1-, 3-, and 6-month follow-ups, and $30 as bonus if all assessments were completed] " See 4a for eligibility requirements.

5-viii) Mode of delivery, features/functionalities/components of the intervention and comparator, and the theoretical framework

We reference the program development and formative evaluation paper that includes much of this information:"Moore SK, Grabinski M, Bessen S, Borodovsky JT, Marsch LA. Web-based prescription opioid abuse prevention for adolescents: program development and formative evaluation. JMIR Form Dev 2019;3(31):e12389. PMID:31325289"<br><br>Detail on theoretical frameworks are included as follows:<br><br>"... The scientific evidence underpinning the Pop4Teen's program content comes from research on identified risk factors for prescription opioid use among youth [27], computer-delivered interventions (CDIs), and [28] computer-assisted instruction (CAI) technology [29].<br>...unique compared to other programs as it is the first of its kind to incorporate knowledge about risk factors for prescription opioid misuse...In addition to a focus on the specific risk factors associated with POs, the interactive, activity-oriented Pop4Teens program is rooted in the prevention science literature... "		
**5-ix) Describe use parameters**		
**5-x) Clarify the level of human involvement**		
**5-xi) Report any prompts/reminders used**		
- computer-generated links to assessments sent automatically at the specified assessment timepoints based on individual participant start dates, also, computer-generated prompts to remind people to complete assessments within specified time windows. A research assistant was available by email to trouble-shoot problems. No other prompts/reminders used.		
**5-xii) Describe any co-interventions (incl. training/support)**		
There were no co-interventions including training. However, a research assistant was available for support by email or phone if a participant had questions.		
**6a) CONSORT: Completely defined pre-specified primary and secondary outcome measures, including how and when they were assessed**		
"Behavioral assessments accessible by links emailed to participants were completed at baseline and follow-up time points (1-, 3- and 6-months). At baseline only, participants completed a form measuring basic demographic and substance use history... At the 1-month time point only, participants completed the program feedback survey..."<br><br>Primary and secondary outcome measures completely defined.		
**6a-i) Online questionnaires: describe if they were validated for online use and apply CHERRIES items to describe how the questionnaires were designed/deployed**		
**6a-ii) Describe whether and how "use" (including intensity of use/dosage) was defined/measured/monitored**		
"Assessment of Usage Data: We quantified usage statistics of the web-based program by assessing the extent of module completion...."		
**6a-iii) Describe whether, how, and when qualitative feedback from participants was obtained**		
**6b) CONSORT: Any changes to trial outcomes after the trial commenced, with reasons**		
All data were collected online (see verbiage above in 4a-ii).		
**7a) CONSORT: How sample size was determined**		
**7a-i) Describe whether and how expected attrition was taken into account when calculating the sample size**		
**7b) CONSORT: When applicable, explanation of any interim analyses and stopping guidelines**		
"Behavioral assessments accessible by links emailed to participants were completed at baseline and follow-up time points (1-, 3- and 6-months). At baseline only, participants completed a form measuring basic demographic and substance use history... At the 1-month time point only, participants completed the program feedback survey..."<br><br>Primary and secondary outcome measures completely defined.		
**8a) CONSORT: Method used to generate the random allocation sequence**		
"Participants were assigned at random using a non-linear random number generator based on an AES block cipher in counter mode to either the experimental or active control condition."		
**8b) CONSORT: Type of randomisation; details of any restriction (such as blocking and block size)**		
There was no blocking.		
**9) CONSORT: Mechanism used to implement the random allocation sequence (such as sequentially numbered containers), describing any steps taken to conceal the sequence until interventions were assigned**		
Randomization was done on strictly on computers using the algorithm described above.		
**10) CONSORT: Who generated the random allocation sequence, who enrolled participants, and who assigned participants to interventions**		
As soon as someone signed up, the server assigned participants. No human intervention.		
**11a) CONSORT: Blinding - If done, who was blinded after assignment to interventions (for example, participants, care providers, those assessing outcomes) and how**		
**11a-i) Specify who was blinded, and who wasn't**		
There was no blinding in this study.		
**11a-ii) Discuss e.g., whether participants knew which intervention was the "intervention of interest" and which one was the "comparator"**		
**11b) CONSORT: If relevant, description of the similarity of interventions**		
Our description of the two interventions underscores the similarity: science-based information about opioids delivered via a web-based platform and including true stories of youth who have suffered due to drug use, information about consequences of drug use, videos and quiz(zes).		
**12a) CONSORT: Statistical methods used to compare groups for primary and secondary outcomes**		
"...Groups were compared for differences on demographics and other characteristics using t-tests, Wilcoxon Rank Sum Tests for non-normal variables and chi-square tests for categorical measures. Program feedback (collected at 1M) was also compared via t-tests. Primary analyses included all participants randomized to a study condition independent of early dropout, consistent with an intent-to-treat approach to clinical trials [62]...Outcome measures were evaluated in separate analyses. Data from primary outcomes were evaluated using mixed-effect models, which allows for non-independent data within individual and are robust to incomplete data. All participants with any baseline or follow-up assessments were included in the outcome analyses. In addition to the study condition indicator, these models included, in the fixed effects portion of the model, a main effect for time as well as a two-way interaction between study condition and time. Before evaluating the comparative effectiveness of P4T, each program's effectiveness in impacting primary outcome variables without comparison is reviewed as neither program has previously been evaluated. The key estimate from this model is the two-way interaction effect, which indicates whether changes in outcome over time are different for the two conditions."		
**12a-i) Imputation techniques to deal with attrition / missing values**		
"Primary analyses included all participants randomized to a study condition independent of early dropout, consistent with an intent-to-treat approach to clinical trials."		
**12b) CONSORT: Methods for additional analyses, such as subgroup analyses and adjusted analyses**		
We present model-estimated means for each outcome for people who answered Y or N to friend used opioids, reported poor mental health, and reported prescribed opioid in past.		
RESULTS		
**13a) CONSORT: For each group, the numbers of participants who were randomly assigned, received intended treatment, and were analysed for the primary outcome**		
All included in our Consort Diagram.		
**13b) CONSORT: For each group, losses and exclusions after randomisation, together with reasons**		
See Consort Diagram. Reasons not included as we were not in contact with participants following 'drop out.'		
**13b-i) Attrition diagram**		
**14a) CONSORT: Dates defining the periods of recruitment and follow-up**		
"...sample of youth... was recruited between June 2017 and February 2018 and data collection was completed in September 2018. "		
**14a-i) Indicate if critical "secular events" fell into the study period**		
**14b) CONSORT: Why the trial ended or was stopped (early)**		
The trial was not stopped early.		
**15) CONSORT: A table showing baseline demographic and clinical characteristics for each group**		
See Table 1.		
**15-i) Report demographics associated with digital divide issues**		
Demographic data include age, education, and gender.		
**16a) CONSORT: For each group, number of participants (denominator) included in each analysis and whether the analysis was by original assigned groups**		
**16-i) Report multiple "denominators" and provide definitions**		

In Table 2 we include the following detail: "N's across time points by group: P4T: B (205), 1M (172), 3M (155), 6M (144): JTT: B (200), 1M (172), 3M (154), 6M (139)"		
**16-ii) Primary analysis should be intent-to-treat**		
"...consistent with an intent-to-treat approach to clinical trials [62]...Outcome measures were evaluated in separate analyses..."		
**17a) CONSORT: For each primary and secondary outcome, results for each group, and the estimated effect size and its precision (such as 95% confidence interval)**		
Se Table 2 for Detailed Means. See Table 3 for Odds Ratios for each outcome and program comparison.		
**17a-i) Presentation of process outcomes such as metrics of use and intensity of use**		
**17b) CONSORT: For binary outcomes, presentation of both absolute and relative effect sizes is recommended**		
We do not include this level of detail.		
**18) CONSORT: Results of any other analyses performed, including subgroup analyses and adjusted analyses, distinguishing pre-specified from exploratory**		
See Table 4 that includes results from subgroup analyses pre-specified based on literature regarding youth at increased risk of misuse of prescription opioids.		
**18-i) Subgroup analysis of comparing only users**		
**19) CONSORT: All important harms or unintended effects in each group**		
No harms or unintended effects.		
**19-i) Include privacy breaches, technical problems**		
**19-ii) Include qualitative feedback from participants or observations from staff/researchers**		
DISCUSSION		
**20) CONSORT: Trial limitations, addressing sources of potential bias, imprecision, multiplicity of analyses**		
**20-i) Typical limitations in ehealth trials**		
We could do a better job of articulating limitations. We do address some limitations of the sample in the following:<br><br>"...direction for future research might include an enhanced approach to target at-risk subgroups (e.g., PMH, FNM, MPU) as the pattern of findings point to different risk factors based on the different risk profiles... due to the ceiling effect we found with respect to perceived physical and other risks and the floor effect for intentions to use, it may be useful to recruit youth who may already be experimenting and/or who endorse increased sensation seeking or self-medicating profiles [77]... Future research may also require an enhanced approach to target the misuse of synthetic opioids like illicit fentanyl by youth... "		
**21) CONSORT: Generalisability (external validity, applicability) of the trial findings**		
**21-i) Generalizability to other populations**		
**21-ii) Discuss if there were elements in the RCT that would be different in a routine application setting**		
**22) CONSORT: Interpretation consistent with results, balancing benefits and harms, and considering other relevant evidence**		
**22-i) Restate study questions and summarize the answers suggested by the data, starting with primary outcomes and process outcomes (use)**		
Our discussion presents the primary findings in the context of the stated aims.		
**22-ii) Highlight unanswered new questions, suggest future research**		
Other information		
**23) CONSORT: Registration number and name of trial registry**		
Trial Registration. ClinicalTrials.gov NCT02737696		
**24) CONSORT: Where the full trial protocol can be accessed, if available**		
"Moore SK, Grabinski M, Bessen S, Borodovsky JT, Marsch LA. Web-based prescription opioid abuse prevention for adolescents: program development and formative evaluation. JMIR Form Dev 2019;3(31):e12389. PMID:31325289"		
**25) CONSORT: Sources of funding and other support (such as supply of drugs), role of funders**		
2R42DA023731 – 02 (NIH, NIDA)		
**X26-i) Comment on ethics committee approval**		
**x26-ii) Outline informed consent procedures**		
**X26-iii) Safety and security procedures**		
**X27-i) State the relation of the study team towards the system being evaluated**		
We did not include a conflict of interest statement in the paper. However, we plan to do so if given the opportunity to revise.		
