# Supplementary material for: Evaluating the Effectiveness of a Web-Based Program (POP4Teens) to Prevent Prescription Opioid Misuse Among Adolescents: Randomized Controlled Trial
Source: JMIR Public Health Surveill. 2021 Feb 25;7(2):e18487. doi: 10.2196/18487 (PMC8128362; doi:10.2196/18487)
Supplement: Multimedia Appendix 2 [file publichealth_v7i2e18487_app2.docx]

**Multimedia Appendix 2.** Primary and secondary outcome (means)^a^.

| Outcome and time point^b^ | | | P4T^c^ | JTT^d^ | Sig.^e^ |
| --- | --- | --- | --- | --- | --- |
|  | | | Mean (SD) | Mean (SD) |  |
| **Positive expectancies** | | | | | |
|  | **Feel good** | | | | |
|  |  | Baseline | 2.67 (1.13) | 2.66 (1.01) | NA |
|  |  | 1 month | 2.41 (1.11) | 2.42 (1.16) | —^f^ |
|  |  | 3 months | 2.39 (1.15) | 2.32 (1.13) | — |
|  |  | 6 months | 2.39 (1.17) | 2.38 (1.13) | — |
|  | **Escape problems** | | | | |
|  |  | Baseline | 2.05 (1.05) | 2.15 (1.03) | NA |
|  |  | 1 month | 1.77 (0.94) | 1.81 (0.94) | — |
|  |  | 3 months | 1.81 (0.97) | 1.66 (0.86) | — |
|  |  | 6 months | 1.63 0.91) | 1.69 (0.91) | — |
|  | **Reduce physical pain** | | | | |
|  |  | Baseline | 3.58 (1.06) | 3.48 (1.06) | NA |
|  |  | 1 month | 3.21 (1.29) | 3.08 (1.30) | — |
|  |  | 3 months | 3.03 (1.35) | 3.08 (1.32) | — |
|  |  | 6 months | 3.17 (1.37) | 3.08 (1.40) | — |
|  | **Reduce anxiety** | | | | |
|  |  | Baseline | 2.80 (1.13) | 2.86 (1.06) | NA |
|  |  | 1 month | 2.38 (1.21) | 2.47 (1.17) | — |
|  |  | 3 months | 2.45 (1.23) | 2.37 (1.17) | — |
|  |  | 6 months | 2.32 (1.20) | 2.35 (1.20) | — |
|  | **Reduce sadness** | | | | |
|  |  | Baseline | 2.46 (1.03) | 2.59 (1.01) | NA |
|  |  | 1 month | 2.10 (1.07) | 2.22 (1.06) | — |
|  |  | 3 months | 2.15 (1.10) | 2.18 (1.06) | — |
|  |  | 6 months | 2.07 (1.08) | 2.08 (1.08) | — |
|  | **Improve social situations** | | | | |
|  |  | Baseline | 2.04 (1.03) | 2.02 (0.87) | NA |
|  |  | 1 month | 1.69 (0.98) | 1.81 (0.96) | — |
|  |  | 3 months | 1.76 (0.96) | 1.64 (0.85) | — |
|  |  | 6 months | 1.70 (0.94) | 1.78 (0.98) | — |
|  | **Reduce boredom** | | | | |
|  |  | Baseline | 2.20 (1.16) | 2.19 (1.04) | NA |
|  |  | 1 month | 1.88 (1.03) | 2.04 (1.07) | — |
|  |  | 3 months | 1.93 (1.02) | 1.79 (0.99) | — |
|  |  | 6 months | 1.83 (1.09) | 1.86 (1.01) | — |
|  | **Lose weight** | | | | |
|  |  | Baseline | 2.38 (1.06) | 2.59 (0.91) | NA |
|  |  | 1 month | 2.15 (1.00) | 2.37 (1.09) | — |
|  |  | 3 months | 2.18 (1.07) | 2.37 (1.12) | — |
|  |  | 6 months | 2.20 (1.11) | 2.37 (1.04) | — |
| **Negative expectancies** | | | | | |
|  | **Get in trouble with parents** | | | | |
|  |  | Baseline | 4.54 (0.91) | 4.54 (0.93) | NA |
|  |  | 1 month | 4.62 (0.84) | 4.61 (0.81) | — |
|  |  | 3 months | 4.64 (0.80) | 4.47 (1.05) | — |
|  |  | 6 months | 4.65 (0.74) | 4.57 (0.83) | — |
|  | **Do poorly in school** | | | | |
|  |  | Baseline | 3.87 (1.15) | 4.03 (0.96) | NA |
|  |  | 1 month | 4.20 (0.99) | 4.22 (0.95) | — |
|  |  | 3 months | 4.24 (1.02) | 4.08 (1.05) | ^e^ |
|  |  | 6 months | 4.34 (0.92) | 4.17 (0.92) | ^e^ |
|  | **Spend too much money** | | | | |
|  |  | Baseline | 3.80 (1.13) | 3.89 (1.08) | NA |
|  |  | 1 month | 4.05 (1.06) | 4.20 (1.04) | — |
|  |  | 3 months | 4.23 (1.00) | 4.01 (1.21) | ^e^ |
|  |  | 6 months | 4.19 (0.98) | 4.17 (1.08) | — |
|  | **Feel sick** | | | | |
|  |  | Baseline | 3.78 (0.94) | 3.82 (0.87) | NA |
|  |  | 1 month | 3.99 (1.01) | 4.10 (0.90) | — |
|  |  | 3 months | 4.12 (0.93) | 3.94 (1.06) | ^e^ |
|  |  | 6 months | 4.13 (0.90) | 4.06 (0.89) | — |
|  | **Feel sleepy** | | | | |
|  |  | Baseline | 3.78 (0.94) | 3.82 (0.87) | NA |
|  |  | 1 month | 3.92 (0.98) | 3.93 (0.95) | — |
|  |  | 3 months | 4.10 (0.91) | 3.92 (1.00) | — |
|  |  | 6 months | 4.04 (0.92) | 4.02 (0.92) | — |
|  | **Pass out** | | | | |
|  |  | Baseline | 3.31 (0.95) | 3.36 (0.92) | NA |
|  |  | 1 month | 3.48 (1.03) | 3.56 (0.97) | — |
|  |  | 3 months | 3.71 (1.03) | 3.42 (1.07) | ^e^ |
|  |  | 6 months | 3.64 (1.04) | 3.60 (1.01) | — |
|  | **Overdose** | | | | |
|  |  | Baseline | 2.82 (1.10) | 2.90 (1.15) | NA |
|  |  | 1 month | 3.19 (1.20) | 3.07 (1.18) | — |
|  |  | 3 months | 3.19 (1.24) | 3.07 (1.18) | — |
|  |  | 6 months | 3.19 (1.15) | 3.15 (1.19) | — |
| **Perceived physical risk** | | | | | |
|  | Baseline | | 4.38 (0.83) | 4.32 (0.83) | NA |
|  | 1 month | | 4.55 (0.77) | 4.59 (0.72) | — |
|  | 3 month | | 4.59 (0.62) | 4.53 (0.71) | — |
|  | 6 month | | 4.49 (0.78) | 4.58 (0.71) | — |
|  | **Low perceived physical risk (no risk/slight risk vs great risk/moderate risk/I am not sure)** | | | | |
|  |  | Baseline | 0.06 (0.24) | 0.06 (0.23) | NA |
|  |  | 1 month | 0.03 (0.18) | 0.03 (0.17) | — |
|  |  | 3 months | 0.02 (0.14) | 0.03 (0.18) | — |
|  |  | 6 months | 0.04 (0.20) | 0.04 (0.19) | — |
| **Perceived other risk** | | | | | |
|  | Baseline | | 4.47 (0.71) | 4.36 (0.81) | NA |
|  | 1 month | | 4.59 (0.74) | 4.63 (0.66) | — |
|  | 3 months | | 4.70 (0.63) | 4.58 (0.67) | — |
|  | 6 months | | 4.62 (0.69) | 4.62 (0.65) | — |
|  | **Low perceived "other" risk (no risk/slight risk vs great risk/moderate risk/I am not sure)** | | | | |
|  |  | Baseline | 0.01 (0.12) | 0.05 (0.21) | NA |
|  |  | 1 month | 0.04 (0.20) | 0.02 (0.13) | ^e^ |
|  |  | 3 months | 0.02 (0.14) | 0.03 (0.16) | — |
|  |  | 6 months | 0.03 (0.16) | 0.03 (0.17) | — |
|  | **Knowledge (accuracy, %)** | | | | |
|  |  | Baseline | 0.89 (0.13) | 0.88 (0.15) | NA |
|  |  | 1 month | 0.93 (0.13) | 0.87 (0.18) | ^e^ |
|  |  | 3 months | 0.94 (0.09) | 0.89 (0.14) | ^e^ |
|  |  | 6 months | 0.93 (0.13) | 0.90 (0.13) | — |
| **Skills** | | | | | |
|  | **Difficult to refuse offer** | | | | |
|  |  | Baseline | 1.62 (0.87) | 1.54 (0.79) | NA |
|  |  | 1 month | 1.36 (0.70) | 1.42 (0.80) | ^e^ |
|  |  | 3 month | 1.35 (0.64) | 1.32 (0.63) | — |
|  |  | 6 month | 1.28 (0.66) | 1.32 (0.72) | — |
|  | **Able to refuse offer** | | | | |
|  |  | Baseline | 1.47 (0.75) | 1.46 (0.74) | NA |
|  |  | 1 month | 1.32 (0.56) | 1.34 (0.70) | — |
|  |  | 3 months | 1.36 (0.79) | 1.31 (0.63) | — |
|  |  | 6 months | 1.24 (0.57) | 1.21 (0.50) | — |
|  | **Difficult to refuse request for prescription opioid** | | | | |
|  |  | Baseline | 1.60 (0.89) | 1.63 (0.92) | NA |
|  |  | 1 month | 1.32 (0.56) | 1.34 (0.70) | — |
|  |  | 3 months | 1.43 (0.75) | 1.41 (0.78) | — |
|  |  | 6 months | 1.35 (0.73) | 1.29 (0.72) | — |
|  | **Able to refuse request for prescription opioid** | | | | |
|  |  | Baseline | 1.51 (0.84) | 1.53 (0.82) | NA |
|  |  | 1 month | 1.31 (0.64) | 1.30 (0.60) | — |
|  |  | 3 months | 1.30 (0.63) | 1.34 (0.63) | — |
|  |  | 6 months | 1.26 (0.57) | 1.26 (0.65) | — |
|  | **Intention to use prescription opioids within 12 months** | | | | |
|  |  | Baseline | 1.49 (0.73) | 1.49 (0.71) | NA |
|  |  | 1 month | 1.31 (0.61) | 1.27 (0.60) | — |
|  |  | 3 months | 1.33 (0.68) | 1.31 (0.63) | — |
|  |  | 6 months | 1.37 (0.68) | 1.32 (0.62) | — |
| **Feedback survey items (1 month)** | | | | | |
|  | How interesting? | | 7.37 (1.88) | 7.09 (2.26) | — |
|  | How useful? | | 7.87 (2.16) | 7.46 (2.16) | — |
|  | How much new information? | | 7.27 (2.48) | 7.05 (2.46) | — |
|  | How easy to use? | | 9.33 (1.22) | 8.31 (2.05) | ^e^ |
|  | How much applies to your life? | | 5.92 (2.66) | 5.38 (2.66) | ^e^ |
|  | How comparable with previous education/training? | | 8.04 (1.91) | 7.98 (1.88) | — |
|  | How useful as part of drug abuse prevention programs? | | 8.24 (1.95) | 8.02 (2.02) | — |
|  | How likely to help teens change behavior? | | 6.87 (2.35) | 6.68 (2.23) | — |
|  | How much liked the laptop, phone, tablet for use? | | 8.94 (1.60) | 8.34 (2.06) | ^e^ |
|  | How much liked videos? | | 6.92 (2.37) | 6.93 (2.24) | — |
|  | How much liked quizzes? | | 7.27 (2.37) | 7.08 (2.37) | — |

^a^Likert item anchors/values: expectancies: strongly disagree=1, disagree=2, not sure=3, agree=4, strongly agree=5; risks: no risk=1, not sure=3, great risk=5; skills/difficulty: very easy=1, easy=2, not sure=3, hard=4, very hard=5; skills/ability: definitely would=1, would=2, not sure=3, would not=4, definitely would not=5; intentions: definitely will not=1, will not=2, not sure=3, will=4, definitely will=5.

^b^N across time points by group: P4T: baseline (205), 1 month (173), 3 month (155), 6 month (144); JTT: baseline (200), 1 month (172), 3 month (154), 6 month (139).

^c^P4T: POP4Teens.

^d^JTT: JustThinkTwice.

^e^Significance at *P*≤.05 level.

^f^A dash (—) is used to indicate non-significant findings.
