# Supplementary material for: Evaluating the Effectiveness of a Web-Based Program (POP4Teens) to Prevent Prescription Opioid Misuse Among Adolescents: Randomized Controlled Trial
Source: JMIR Public Health Surveill. 2021 Feb 25;7(2):e18487. doi: 10.2196/18487 (PMC8128362; doi:10.2196/18487)
Supplement: Multimedia Appendix 3 [file publichealth_v7i2e18487_app3.docx]

**Multimedia Appendix 3.** Primary and secondary outcome (mixed models)**.**

|  | | | | | | | | |
| --- | --- | --- | --- | --- | --- | --- | --- | --- |
|  |  | **Independent Program Effectiveness** | | | | | | **Comparative**  **Program**  **Effectiveness** |
|  |  | P4T | | | JTT | | |  |
|  |  | Mean Estimate or  OR | SE  or  CI | *p*-value | Mean Estimate or  OR | SE  or  CI | *p*-value | *p*-value |
| **POSITIVE EXPECTANCIES** |  |  |  |  |  |  |  |  |
| Feel good | 1 M | -0.28 | 0.09 | —^a^ | -0.21 | 0.09 | —^b^ | 0.58 |
|  | 3 M | -0.31 | 0.09 | —^a^ | -0.31 | 0.09 | —^c^ | 0.99 |
|  | 6 M | -0.28 | 0.09 | —^a^ | -0.23 | 0.09 | —^b^ | 0.69 |
| Escape problems | 1 M | -0.29 | 0.08 | —^a^ | -0.31 | 0.08 | —^d^ | 0.84 |
|  | 3 M | -0.28 | 0.08 | —^a^ | -0.43 | 0.08 | —^d^ | 0.19 |
|  | 6 M | -0.41 | 0.08 | —^d^ | -0.39 | 0.09 | —^d^ | 0.88 |
| Reduce physical pain | 1 M | -0.41 | 0.09 | —^d^ | -0.39 | 0.09 | —^d^ | 0.88 |
|  | 3 M | -0.57 | 0.10 | —^d^ | -0.42 | 0.10 | —^d^ | 0.28 |
|  | 6 M | -0.43 | 0.10 | —^d^ | -0.38 | 0.10 | —^a^ | 0.74 |
| Reduce anxiety | 1 M | -0.45 | 0.09 | —^d^ | -0.37 | 0.09 | —^d^ | 0.57 |
|  | 3 M | -0.37 | 0.09 | —^d^ | -0.46 | 0.09 | —^d^ | 0.47 |
|  | 6 M | -0.49 | 0.10 | —^d^ | -0.43 | 0.10 | —^d^ | 0.7 |
| Reduce sadness | 1 M | -0.37 | 0.08 | —^d^ | -0.35 | 0.08 | —^d^ | 0.91 |
|  | 3 M | -0.31 | 0.09 | —^c^ | -0.34 | 0.09 | —^d^ | 0.8 |
|  | 6 M | -0.37 | 0.09 | —^d^ | -0.40 | 0.09 | —^d^ | 0.84 |
| Improve social situations | 1 M | -0.32 | 0.08 | —^d^ | -0.19 | 0.08 | —^b^ | 0.24 |
|  | 3 M | -0.28 | 0.08 | —^c^ | -0.32 | 0.08 | —^c^ | 0.71 |
|  | 6 M | -0.32 | 0.08 | —^c^ | -0.17 | 0.09 | —^b^ | 0.22 |
| Reduce boredom | 1 M | -0.29 | 0.08 | —^c^ | -0.13 | 0.08 | **0.11** | 0.16 |
|  | 3 M | -0.27 | 0.08 | —^c^ | -0.35 | 0.08 | —^d^ | 0.47 |
|  | 6 M | -0.36 | 0.09 | —^d^ | -0.23 | 0.09 | —^b^ | 0.3 |
| Lose weight | 1 M | -0.24 | 0.09 | —^b^ | -0.20 | 0.09 | —^b^ | 0.77 |
|  | 3 M | -0.21 | 0.09 | —^b^ | -0.21 | 0.09 | —^b^ | 1 |
|  | 6 M | -0.18 | 0.09 | —^b^ | -0.17 | 0.09 | **0.06** | 0.96 |
| **NEGATIVE EXPECTANCIES** |  |  |  |  |  |  |  |  |
| Get in trouble with parents | 1 M | 0.08 | 0.08 | **0.30** | 0.07 | 0.08 | **0.35** | 0.95 |
|  | 3 M | 0.10 | 0.08 | **0.20** | -0.08 | 0.08 | **0.32** | 0.11 |
|  | 6 M | 0.10 | 0.08 | **0.22** | 0.01 | 0.08 | **0.88** | 0.46 |
| Do poorly in school | 1 M | 0.33 | 0.08 | —^d^ | 0.18 | 0.08 | —^b^ | 0.2 |
|  | 3 M | 0.36 | 0.09 | —^d^ | 0.02 | 0.09 | **0.82** | 0.01 |
|  | 6 M | 0.45 | 0.09 | —^d^ | 0.11 | 0.09 | **0.23** | 0.01 |
| Spend too much money | 1 M | 0.24 | 0.09 | —^b^ | 0.30 | 0.09 | —^c^ | 0.64 |
|  | 3 M | 0.44 | 0.09 | —^d^ | 0.10 | 0.09 | **0.28** | 0.01 |
|  | 6 M | 0.39 | 0.09 | —^d^ | 0.25 | 0.09 | —^b^ | 0.31 |
| Feel sick | 1 M | 0.22 | 0.08 | —^c^ | 0.27 | 0.08 | —^c^ | 0.63 |
|  | 3 M | 0.34 | 0.08 | —^d^ | 0.10 | 0.08 | **0.24** | 0.03 |
|  | 6 M | 0.35 | 0.08 | —^d^ | 0.21 | 0.08 | —^b^ | 0.25 |
| Feel sleepy | 1 M | 0.07 | 0.08 | **0.41** | 0.21 | 0.08 | —^b^ | 0.21 |
|  | 3 M | 0.27 | 0.08 | —^c^ | 0.19 | 0.08 | —^b^ | 0.48 |
|  | 6 M | 0.18 | 0.08 | —^b^ | 0.27 | 0.09 | —^c^ | 0.46 |
| Pass out | 1 M | 0.17 | 0.09 | —^b^ | 0.21 | 0.09 | —^b^ | 0.79 |
|  | 3 M | 0.41 | 0.09 | —^d^ | 0.05 | 0.09 | **0.57** | 0 |
|  | 6 M | 0.32 | 0.09 | —^c^ | 0.23 | 0.09 | —^b^ | 0.49 |
| Overdose | 1 M | 0.38 | 0.09 | —^d^ | 0.18 | 0.09 | —^b^ | 0.1 |
|  | 3 M | 0.39 | 0.09 | —^d^ | 0.18 | 0.09 | —^b^ | 0.11 |
|  | 6 M | 0.32 | 0.09 | —^c^ | 0.24 | 0.09 | —^b^ | 0.54 |
| **PERCEIVED**  **PHYSICAL RISK** | 1 M | 0.17 | 0.06 | —^b^ | 0.27 | 0.06 | —^d^ | 0.26 |
|  | 3 M | 0.22 | 0.07 | —^c^ | 0.18 | 0.07 | —^b^ | 0.66 |
|  | 6 M | 0.10 | 0.07 | **0.14** | 0.23 | 0.07 | —^c^ | 0.18 |
| Low perceived physical risk (No risk/Slight risk vs. Great risk/Moderate risk/I am not sure)  Chi-Square | 1 M | 0.57 | (0.27,  1.21) | **0.14** | 0.50 | (0.18, 1.41) | **0.19** | 0.85 |
|  | 3 M | 0.28 | (0.08, 0.96) | —^b^ | 0.57 | (0.22, 1.49) | **0.25** | 0.37 |
|  | 6 M | 0.68 | (0.28, 1.67) | **0.40** | 0.68 | (0.25, 1.84) | **0.45** | 1 |
| **PERCEIVED**  **OTHER RISK** | 1 M | 0.12 | 0.06 | **0.06** | 0.26 | 0.06 | —^d^ | 0.08 |
|  | 3 M | 0.22 | 0.06 | —^c^ | 0.18 | 0.06 | —^c^ | 0.66 |
|  | 6 M | 0.12 | 0.06 | **0.06** | 0.24 | 0.07 | —^c^ | 0.21 |
| Low perceived "other" risk (No risk/Slight risk vs. Great risk/Moderate risk/I am not sure)  Chi-Square | 1 M | 2.87 | (0.73, 11.26) | **0.13** | 0.37 | (0.11, 1.27) | **0.11** | 0.03 |
|  | 3 M | 1.30 | (0.25, 6.63) | **0.75** | 0.62 | (0.24, 1.57) | **0.31** | 0.44 |
|  | 6 M | 1.97 | (0.44, 8.89) | **0.38** | 0.65 | (0.20, 2.13) | **0.48** | 0.26 |
| **KNOWLEDGE** | 1 M | 0.59 | 0.14 | —^d^ | -0.07 | 0.14 | **0.61** | 0 |
|  | 3 M | 0.65 | 0.14 | —^d^ | -0.01 | 0.14 | **0.96** | 0 |
|  | 6 M | 0.58 | 0.15 | —^d^ | 0.19 | 0.15 | **0.21** | 0.06 |
| **SKILLS** |  |  |  |  |  |  |  |  |
| Difficult to refuse offer | 1 M | -0.25 | 0.06 | —^d^ | -0.09 | 0.06 | **0.14** | 0.05 |
|  | 3 M | -0.28 | 0.06 | —^d^ | -0.17 | 0.06 | —^b^ | 0.23 |
|  | 6 M | -0.33 | 0.06 | —^d^ | -0.16 | 0.06 | —^b^ | 0.06 |
| Able to refuse offer | 1 M | -0.13 | 0.05 | —^b^ | -0.11 | 0.05 | —^b^ | 0.73 |
|  | 3 M | -0.11 | 0.06 | **0.06** | -0.14 | 0.06 | —^b^ | 0.73 |
|  | 6 M | -0.21 | 0.06 | —^c^ | -0.19 | 0.06 | —^c^ | 0.83 |
| Difficult to refuse request for prescription opioid | 1 M | -0.29 | 0.06 | —^d^ | -0.25 | 0.06 | —^d^ | 0.69 |
|  | 3 M | -0.16 | 0.06 | —^b^ | -0.20 | 0.06 | —^c^ | 0.66 |
|  | 6 M | -0.23 | 0.07 | —^c^ | -0.33 | 0.07 | —^d^ | 0.29 |
| Able to refuse request for prescription opioid | 1 M | -0.20 | 0.06 | —^c^ | -0.23 | 0.06 | —^d^ | 0.73 |
|  | 3 M | -0.20 | 0.06 | —^c^ | -0.19 | 0.06 | —^c^ | 0.84 |
|  | 6 M | -0.25 | 0.06 | —^d^ | -0.26 | 0.06 | —^d^ | 0.91 |
| **INTENTION TO USE PO’s w/in 12 MONTHS** | 1 M | -0.17 | 0.05 | —^c^ | -0.20 | 0.05 | —^c^ | 0.68 |
|  | 3 M | -0.15 | 0.06 | —^b^ | -0.16 | 0.06 | —^b^ | 0.99 |
|  | 6 M | -0.11 | 0.06 | —^b^ | -0.14 | 0.06 | —^b^ | 0.75 |
| Baseline = reference for 1-, 3- and 6-M(month) observations | | | | | | | | |

^a^*P*<.001.

^b^*P*≤.05.

^c^*P*<.01.

^d^*P*<.0001.
